# Differences in collembola species assemblages (Arthropoda) between spoil tips and surrounding environments are dependent on vegetation development

**DOI:** 10.1038/s41598-018-36315-1

**Published:** 2018-12-24

**Authors:** Benoit Vanhée, Cédric Devigne

**Affiliations:** Equipe Ecologie & Biodiversité, Unité de Recherche Smart & Sustainable Cities, Faculté de Gestion Economie & Sciences, Université Catholique de Lille, 60 Bvd Vauban, 59016 Lille cedex, France

## Abstract

Spoil tip production is one of the most extreme means of soil destruction, replacing the native soil with a coarse substrate. In this paper, we aim to determine the colonization of soil biota in new substrates, using collembola assemblages as an indicator. In Northern France, we sampled collembola communities in 11 coal mine spoil tips and their surroundings divided in four stages of vegetation development: bare soil, meadow, shrub and tree covers. We demonstrated that collembola assemblages of spoil tips were different from those observed in the surrounding native soil. Collembola communities on bare soil were characterized by pioneer (based on the *Indval* index) or exotic species (new in Northern France). However, homogenization occurred with development of vegetation cover. Indeed, our data showed no difference in springtail diversity between spoil tips and their corresponding environments regarding the tree vegetation cover. Using the *Indval* method, we defined pioneer, colonizing, opportunist or stenoecious species as a function of substrate affinities. Using the same method, we defined specialists, elective, preferring or indifferent species as a function of vegetation cover affinities, showing similarities with previously published surveys. Hence, our results were obtained by a focused analysis of species and their particularity. Finally, we discussed the interest in and the complementarity between the species analysis approach and the methodology dealing with functional traits and of its importance in the decision process of restoration and/or conservation of nature.

## Introduction

The World has entered the Anthropocene period^1^ characterised by an increase in urbanization and industrialization, resulting in major environmental changes both at local and global levels. The consequences are already visible on the atmosphere^2^, water^3^ and ecosystem functioning^4^. Soil is not exempt. For example, the biggest schist deposit in Loos-en-Gohelle now replaces at least 90 ha of native soil^5^. As a consequence, around 250 spoil tips in the coal mining industrial areas have transformed over the course of 200 years the landscape of Northern France with their conical or flattened shapes. Spoil tips are accumulations of schist wastes, due to the soil exploitation by mining activities, and visually and chemically pollute the landscape. However, the establishment of spoil tips can also be considered as a new biotope without a functional soil *sensu* Doran^6^. Two complementary approaches could be initiated to reclaim sites: restoration ecology and/or conservation biology^7,8^. For soil restoration, the objective is to maintain the functional ecology, providing essential functions and ecosystem services. In light of this, the functional diversity approach must be promoted^9^, with an interest on functional traits rather than species^10^. However, for soil biodiversity conservation, environmental actions should promote the importance of local, natural heritage and hence should consider the site effect on species composition. Both priorities would benefit from increased knowledge of biotic communities’ and species’ resilience despite local change^11,12^.

On a regional level, coal tips present a pedological break in the environment and appear to be a schist island in the middle of an alluvial clay limestone ocean. Therefore, numerous ecological factors differ locally between spoil tips and their surrounding environment such as soil particles, acidity, temperature, texture, carbon and nitrogen availabilities^13–18^. These variations impact the observed biodiversity in functional or species diversity. Moreover, differences in relief (impacting exposition) or the development of vegetation cover on coal tips also influence colonization, resulting in distinctive soil fauna communities^19^. Indeed, because initial schist deposits come from geological sub-soil^20^, spoil tips present a rare opportunity to follow the process of natural soil colonization of a new technosoil^21–23^. It is possible to observe the successive formation of vegetation covers from bare soil to forest. They are most likely colonized by soil fauna from surrounding landscape^23–25^. In a landscape dominated by intensive agricultural practices or urban environments^26^, spoil tips are becoming sanctuaries for biodiversity. Overall, those natural reserves are well known for the presence of flora^18,26–28^, as well as fungi^29–32^ and epigeic macrofauna^33–39^, although those were poorly surveyed.

The colonization of artificial soil by fauna has been less studied^23,40^. Firstly, despite the fact that artificial soil is originally made with only one strata, fauna differs along a deep gradient during the pedogenesis process and vegetation growth^41,42^. Moreover, strong interactions and feedback loops occur between vegetation and soil fauna, adding differentiation factors and complex linkages in these growing ecosystems^43–45^. Secondly, even if the dispersal abilities of animals could interfere with the occurrence of species^46,47^, no study has addressed the topic of fauna colonization on artificial soil such as spoil tips by the surrounding fauna (but read^48^).

Collembola are small arthropods, playing an essential role in humus formation by actively participating in leaf litter disintegration^49,50^. Moreover they disseminate micro-organisms and are food sources for many predators^49^. Because of their importance in soil functioning^51^, their high abundance and diversity^52^ and their variable sensitivity to soil disturbance^53^, Collembola are considered to be good bioindicators of soil quality^54–57^. Thus, they are used to estimate the soil biodiversity as well as the human impact on such biodiversity^57^. Collembola assemblage recolonization after perturbation has been well studied in native soil^58–60^. Usually, collembola composition between vegetation strata cover^61^ or humus layer^62^ is compared, increasing our knowledge of collembola communities in natural conditions^63^, in urban environments^64,65^ and in industrial landscapes^40^. Nevertheless, the overall effect on the dynamic of collembola communities resulting from environmental changes (e.g. progressive changes like vegetation growth or a sudden one like substrate modification) remains unknown. Recently, experimental studies tested the adaptation of Collembola to such biotope disturbances^46,66^. Although several articles mention the sterile substrata colonized by fauna, the analysis of collembola assemblage according to proximity between new substrata and their environments have not yet been conducted. Interestingly, collembola colonization did not match their dispersal ability^47^. For example, euedaphic species such as *Megalothorax minimus* or *Mesaphorura yosii* showing low dispersal ability have a faster colonization^46,47,67^. In this respect, dispersal ability predicted by morphological characters is not the only controlling factor for colonization. In this paper, we aim to test the three following hypotheses:

**Hypothesis 1**. The soil physico-chemical properties could impact collembola communities.

**Hypothesis 2**. No collembola assemblage should occur on the bare soil of spoil tips. Indeed, no available resources (coming from leaf litter) could be found on such skeletal soil for collembola detritivores.

**Hypothesis 3**. Succession of vegetation cover on spoil tips should induce collembola assemblages variation. However, similar assemblages are expected with similar vegetation cover, whatever the original substrate was.

## Materials and Methods

### Investigated sites

In Northern France, 250 coal tips are inherited from the industrial development at the beginning of the 19^th^ century. Many of these were abandoned from the mid-nineteenth century until 1990, when the last mine was closed. After mining activity, some coal tips currently present a truly spontaneous and noteworthy vegetation^26,27^ and fauna^38^. The requalification of such industrial wasteland is a key goal and has the honor of being a World Heritage Site for UNESCO^68^. The region has an oceanic climate, with an annual rainfall and temperature averaging 680 mm and 11 °C, respectively. On spoil tips, soils are well drained as a function of the slope and the coarse texture of schist deposit.

We selected 11 spoil tips (Table S1) based on the dominant type of surrounding environment (forested, rural, urban). However, these specificities of landscape were not used in following analysis. All studied spoil tips are found in the industrial region of Northwestern France, between the town of Bruay-la-Buissière and Leforest.

On each spoil tips, Collembola and soil samples were collected on four different vegetation covers with each sample corresponding to one of the four steps of vegetation development: bare, meadow, shrub and forest (Fig. S1). The area was located within 4 distinct phytogeographical territories^69^. The bare soil was the first stage. It was characterized by no vegetation or only a spread vegetation represented by the *Resedo luteae - Rumicetum scutati* Petit 1980 association. The next stages of vegetation cover were either the meadow, represented by the *Dauco-Melilotion* Görs 1966 or the shrub stage with the *Sedo-Scleranthetea* Br.-Bl. 1955 association. These stages improve the soil, paving the way for the next step, generally woodland composed of birch trees (*Betula pendula* Roth)^27^.

Collembola samplings were taken between May 22^th^ 2013 and July 13^th^ 2013. For 10 out of 11 coal tips, bare soil was not find in the nearby surroundings. Therefore, bare coal tip soil samples were compared to samples collected from plowed soils in the surrounding environments of the coal tip T122 at Leforest (the only spoil tip with such surrounding features).

### Field sampling and laboratory procedure

Vegetation and soil conditions were analyzed qualitatively (cover vegetation, soil texture) and quantitatively (physicochemical features). For the abiotic charaterization, 147 samples of coal tip soils and 191 samples of soil from the peripheral environment were analyzed (one of samples taken from the meadow category was lost). With 3 samples per station, the bare, meadow, shrub and forest soils were represented respectively by 14, 12, 3 and 20 stations in coal tips and 7, 34, 7 and 16 stations in the surrounding environment.

Soil analyses were carried out based on 8 parameters: clay (<2 µm), fine silt (2 to 20 µm), coarse silt (20 to 50 µm), fine sand (50 to 200 µm), coarse sand (200 to 2000 µm) (NF X31-107), organic matter measured by organic carbon content (Anne method, NF ISO 14235), and nitrogen determination by combustion (Dumas method, NF ISO 13878), and water pH (NF ISO 10390). Graphic representation was performed with the soiltexture package from R^70^. Mann-Whitney test was performed to compare spoil tip and peripheral environment.

To analyze collembola communities, 5 soil core samples spaced 5 m apart were collected at each station. Each soil core sample consisted of 1 dm^3^ of soil collected with an adapted auger (Ø 8.5 cm and 11.5 cm length) capable of preventing escape of fast-moving arthropods. We obtained 405 samples from 81 stations, including 37 spoil tip stations (ST) and 44 peripheral environment stations (PE) (Table S1). For each sample, collembola communities were extracted from the soil over a 10-day period by dry funnel method using a selfmade Berlese-Tullgren funnel^71^. Collembola were conserved in 70% ethyl alcohol until identification. Species identification of Collembola was performed using a phase light contrast microscope at 400–600 x magnification of animals pre-treated in a warm lactic acid bath. In case of large or dark specimens, 10% potassium hydroxide (KOH*aq*) buffer was used to make them translucent. Identification was performed using the synoptic keys^72–78^.

### Taxonomical information

For the species *Cryptopygus thermophilus* (Axelson 1900) and *Cryptopygus bipunctatus* (Axelson 1903), Rusek’s advice^79^ was followed by naming them *Hemisotoma thermophila* (Axelson 1900) and *Proisotomodes bipunctatus* (Axelson 1903), respectively.

Despite the opinion of Hopkin^76^, *Protaphorura octopunctata* (Tullberg 1876) was accepted in our study as a valid taxa, according to the discovery of a stable population corresponding to the Pomorski & Kaprus description^80^.

A population of the Genus *Pseudosinella* with 2 × 5 ocelli required further research (R. Jordana and E. Baquero, pers. comm.) but was temporarily assigned the name of *Pseudosinella* cf. *terricola* (Gisin 1967).

### Community characterization and statistics

To visualize the community, Non-metric MultiDimensional Scaling (NMDS) was performed on the 81 stations based on dissimilarity matrix^81,82^. The dissimilarity matrix was built with Bray-Curtis index based on the abundance of each collembola species (including immature instars). At the opposite of usual correspondence analyses^14,63,83–85^, NMDS does not use the absolute species abundance in communities, but rather a rank order based on dissimilarity index based on this abundance. Therefore, NMDS was used to accommodate for variation of density caused by potential parthenogenesis in a population^86^ or aggregation in some species^87–89^ (but read^90^). This resulted in abundance in samples that could be quite variable. We computed 100 runs of the NMDS algorithm with random starting configurations, compared the results (function Procrustes and Protest). The assay was stopped after finding twice a similar minimum stress solution to ensure that a stable solution was reached. We acknowledged a stress test <0.3, i.e. only less than three times out of ten resulted in another graphic representation. All analyses were computed using the “vegan”^91,92^ package (R software^93^). Segregation between distinct collembola assemblages was statistically tested. Analysis of variance and fitting linear models were performed using ADONIS test on Sorensen distance (200 permutations), which is more robust than ANOSIM^92,94^. Thus, the collembola communities were characterized according to the soil substrate (spoil tip vs peripheral environment) and the vegetation cover. This method first allowed the analysis of collembola community differentiation observed on spoil tips from those on the peripheral environment and second to measure the impact of vegetation cover on communities.

### Characterization of species habitat preference

In order to classify species (i) based on their specificity, preference or indifference to a biotope (j), the *IndVal* index^95^ was used. The *A*_*ij*_ ratio was defined as the ratio between the mean abundance of species *sp*_*i*_ in the group *j* sites and the total abundance of species *sp*_*i*_ in all sites. The *B*_*ij*_ ratio was defined as the relative frequency of occurrence of species *i* in the group *j* sites. For each species *i* in each group *j* site (spoil tip or peripheral environment), the product of *A*_*ij*_ and *B*_*ij*_ was defined as followed:

$$IndVa{l}_{ij}={A}_{ij}\times {B}_{ij}\times 100,$$

Where =

$${A}_{ij}=\frac{Nb\,individuals\,of\,i\,in\,the\,sites\,j}{Nb\,individuals\,of\,i\,in\,all\,sites}$$

$$Bij\,=\,\frac{Nb\,sites\,of\,j\,where\,we\,found\,i}{Nb\,sites\,of\,j}$$

*IndVal*_*ij*_ reached its maximum value (100) when species *i* is present in all soil from habitat *j* and absent in soil from all other habitats. Then, two different analyses were performed. The species’ substrate preference was first studied: peripheral environments or spoil tips. Secondly, the species’ preferences for the 4 categories of vegetation cover (bare, meadow, shrub or tree) were compared.

The first step in this approach was to eliminate species with inadequate sample sizes. Thirty-three species were discarded because the sample size was not sufficient for segregation analysis (n < 10 for whole samples). Then, habitat preference studies were performed for 75 species which represented approximately 70% of the species discovered but more than 99% of individuals.

The “*IndVal*” function of “labdsv” package^96^ (R software^93^) was then used to calculate the indVal values. The geographical unit being the station and the number of collected soil samples being five, only the Collembola of those five sample replicates were pooled for the test. Two different analyses were performed.

#### Spoil tip (ST) and peripheral environment (PE) *IndVal* indices

Each geographic location of spoil tips or peripheral environment as random effects was considered. This provided average abundances for each species in both environment. Statistical differences were tested with a t-test.

For the first (ST *vs*. PE test), 5 situations were identified for each species:

- The species was absent or accidentally in the spoil tip with the *IndVal* index significant *p*-value (p < 0.05) for peripheral environment, the stenoecious group^97^,
- The species was absent or accidentally in the peripheral environment with the *IndVal* index significant *p*-value (p < 0.05) for the spoil tip, the pioneer group^97^,
- The *IndVal* index did not show a significant *p*-value, but a binomial test indicated a simple preference for coal tips, the colonizing group,
- The *IndVal* index did not show a significant *p*-value, but a binomial test indicated a simple preference for peripheral environments, the opportunist group,
- No significant difference was demonstrated with the *IndVal* nor with the binomial test, indicating irrelevance of the substrata, the euryecious group^97^.

#### Bare (B), meadow (M), shrub (S) and tree (T) *IndVal* indices

Results for spoil tips and the peripheral environments were pooled for each type of vegetation cover. This provided abundances for each species in each vegetation covers. Statistical differences were tested with a Pearson Chi-squared test.

To test the relationship between species to vegetation cover, four categories were used: exclusive species were defined as significantly dependent of one specific vegetation cover (*IndVal* indice p < 0.05, regardless of vegetation covers number dependent of the presence of the relative species). For all other cases, species were defined as a function of vegetation covers number and binomial tests result. Species were elective for a choice between two vegetation covers (two vegetation covers concerned, *IndVal* indices p ≥ 0.05, binomial test p < 0.05), preferring for a simple preference between three or four vegetation covers (more than two vegetation covers concerned, *IndVal* indices p ≥ 0.05, binomial test p < 0.05). Indifferent species were observed in three or four vegetation cover types without significant differences (more than two vegetation covers concerned, *IndVal* indice p ≥ 0.05, binomial test p ≥ 0.05).

### Measurement of biodiversity

The data used for statistical treatment were the number of individuals in all identified species in the 5 soil samples pooled according to station. The absolute abundance of species was analysed without transformation in relation to the weight of the samples. Classic biodiversity indices quantifying the abundance of the species (e.g. Simpson and Shannon indices) were used. However, because these indices of entropy are abstract concepts, Hill’s formula was also used^98^ $({}^{q}D={(\sum _{s=1}^{S}{p}_{s}^{q})}^{\frac{1}{1-q}})$. This allowed the integration of different indices in a global data visualisation, thanks to each community’s Renyi biodiversity profile^99–101^. *S* represented the species number and *P*_*s*_ the likelihood that a randomly pulled individual belonged to the species. Indeed, Renyi biodiversity profiles showed Hill’s indices for this community: ^0^*D* = *S* (species richness), ^1^*D* = *e*^*H*^ the Shannon index exponential, and ${}^{2}D=\frac{1}{1-E}$ the inverse of the Gini-Simpson concentration indice.

To compare the biodiversity profiles, we used the package “entropart”^102^ (R software^93^).

## Results

### General observations

Of the 405 samples from 81 stations, a total of 11,127 Collembola were identified and classified in 107 species. Among them, 75 species possessed more than 10 individuals and were used in the following collembola community analysis.

In the area of survey, *Parisotoma notabilis*, *Folsomia quadrioculata*, *Isotoma viridis* and *Lepidocyrtus lanuginosus* were the dominant species independently of the station substrate. Despite being specifically found on spoil tips, three more species were also added to the list of dominant species: *Hemisotoma thermophila*, *Pseudosinella cf terricola* and *Folsomides parvulus*.

### Environmental features (Spoil Tips vs. Peripheral Environments)

**Figure 1:**
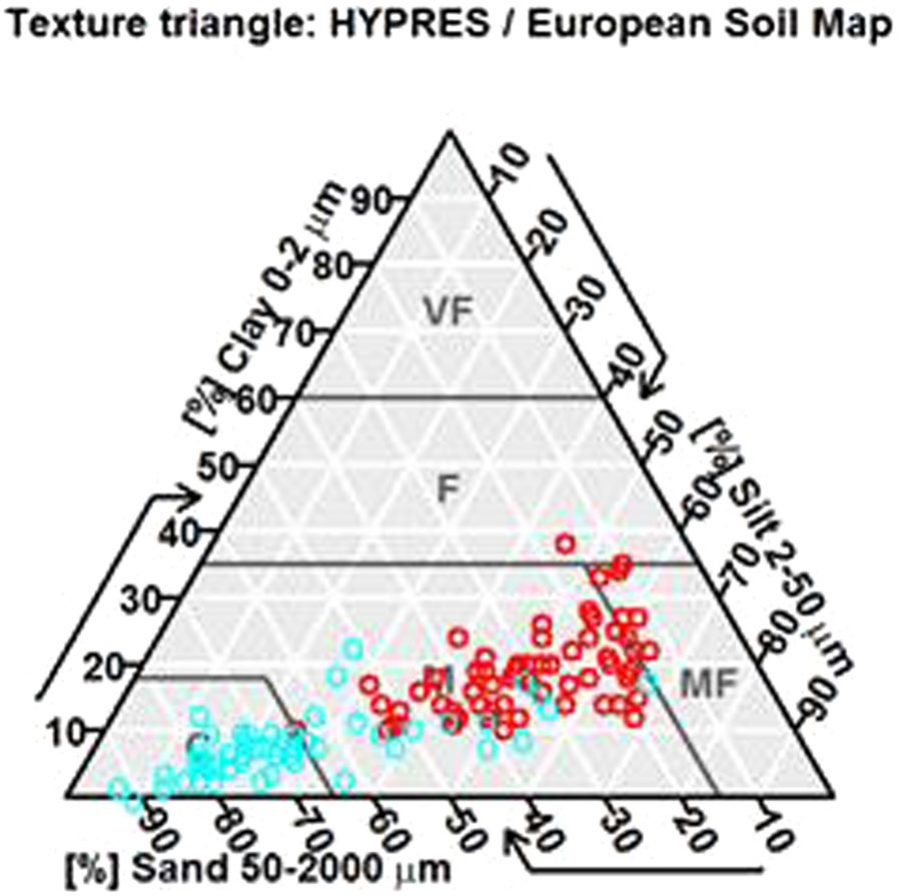
Representation of texture samples of the industrial field of the North of France Spoil tips soils in blue (n = 147); environment soils in red (n = 191). VF = very fine, F = fine, M = medium fine, M = Medium, C = coarse. Analysis performed with package soiltexture in R (Moeys, 2015).

The differences in soil texture between spoil tips and surrounding environments were clear (Fig. 1). Spoil tips were characterized by high sand proportion. At the opposite, peripheral environments were defined by high proportions of silt and clay (Fig. 1). In this respect, spoil tips were dominated by coarse texture whereas the peripheral environment generally had a medium fine texture (Fig. 1). Moreover, significant differences between spoil tips and surrounding soils were observed in all granulometry classes (Table 1, Mann-Whitney test between PE and ST, p < 0.01). Indeed, coarse sand was more abundant in spoil tips than in peripheral environments (Table 1. U = 1462.5, p < 0.001). On the contrary, coarse silt (U = 6434.5, p < 0.001), fine silt (U = 3172.5, p < 0.001), fine sand (U = 11228, p < 0.01) and clay (U = 2661.5, p < 0.001) were all more abundant in the environment than in spoil tips.

**Table 1:** Determination of significant difference between composition of soils of Spoil Tips and Peripheral Environment (ST/PE) and on bare (PE_N/ST_N), meadow (PE_M/ST_M), Shrub ((PE_S/ST_S) and Tree (PE_T/ST_T) cover stations.

Station	N=	Clay (g/kg)	Fine silt (g/kg)	Coarse silt (g/kg)	Fine sand (g/kg)	Coarse sand (g/kg)	Nitrogen (gN/kg)	Org. Matter (g/kg)	pH	C/N
PE	191	191.6 ± 69.5	259.7 ± 116.5	253.3 ± 113.7	161.4 ± 66.6	134.2 ± 114.2	3,38 ± 2,29	53,6 ± 35,9	6,75 ± 1,07	7,26 ± 5,23
ST	147	84.8 ± 49.7	124 ± 46.4	141.9 ± 114.2	138.2 ± 42.3	511.3 ± 173.5	4,12 ± 2,72	68,14 ± 42,11	6,75 ± 1,16	5.86 ± 5.17 *N = 145)*
p		U = 2661.5***	U = 3172.5***	U = 6434.5***	U = 11228**	U = 1462.5***	U = 10437***	U = 10780***	U = 14038NS	U = 10778***
PE_N	21	205.1 ± 51.5	356.3 ± 111.6	154.3 ± 95.1	152.2 ± 74.2	132.1 ± 73.2	1,49 ± 0,49	22,25 ± 5,87	7,78 ± 0,48	12,18 ± 6,69
ST_N	42	56.6 ± 38	107.8 ± 43.1	83.8 ± 45.8	135.4 ± 54.9	616.4 ± 141.5	2,91 ± 1,38	45,22 ± 27,74	7,46 ± 0,86	6,80 ± 6,74
p		U = 15.5***	U = 11.5***	U = 274.5*	U = 405.5NS	U = 15 ***	U = 87***	U = 175***	U = 332NS	U = 175***
PE_M	101	190.1 ± 68.7	247.9 ± 108.1	270.4 ± 103.5	158.7 ± 57.1	133.1 ± 111.2	3,32 ± 2,18	50,87 ± 30,24	6,8 ± 0,87	7,18 ± 5,38
ST_M	36	98.1 ± 48.4	124.1 ± 41.8	189.4 ± 135.5	136.1 ± 31.5	452.6 ± 196.2	3,50 ± 1,23	61,86 ± 32,50	6,83 ± 1	6,73 ± 5,39
p		U = 452 ***	U = 434***	U = 1049.5***	U = 1443.5 NS	U = 352***	U = 1358.5*	U = 1409*	U = 1767.5NS	U = 1648.5NS
PE_S	21	208.9 ± 73.6	249.8 ± 125.4	242.8 ± 106.9	138.9 ± 58.4	159.9 ± 171.1	2,94 ± 1,20	52,96 ± 25,68	7,34 ± 0,60	7,50 ± 3,86
ST_S	9	75 ± 24.5	103.4 ± 35.4	107.2 ± 38	153.3 ± 44.2	561.1 ± 68.1	3,78 ± 1,13	72,58 ± 31,54	7,12 ± 0,54	5,94 ± 3,55
P		U = 10 ***	U = 15***	U = 31.5**	U = 74NS	U = 9***	U = 52.5NS	U = 61NS	U = 79NS	U = 76NS
PE_T	48	181.2 ± 75.5	246.5 ± 115.6	265.2 ± 124.8	180.9 ± 81	126.3 ± 106.4	4,52 ± 2,72	73,38 ± 46	5,96 ± 1,2	5,18 ± 2,9
ST_T	60	98.1 ± 52.6	138.3 ± 48.6	159.3 ± 124.3	139.1 ± 37.9	465.5 ± 157.1	5,4 ± 3,6	87,27 ± 48,16	6,14 ± 1,19	4.62 ± 3.55(N = 58)
p		U = 450.5***	U = 457***	U = 771.5***	U = 957.5**	U = 122***	U = 1171.5NS	U = 1154.5NS	U = 1290NS	U = 1169NS

Mann-Whithney test: NS = Non significant, *pvalue < 0.05, **pvalue < 0.01, ***pvalue < 0.001. Analysis performed with Past 3.0.

Organic matter was also more abundant in spoil tips than in peripheral environments (Table 1. U = 10780, p < 0.001) whereas total nitrogen and C/N ratio were higher in peripheral environments (U = 10778, p < 0.001). Finally, pH values did not differ between spoil tips and peripheral environments and were both slightly basic (U = 14038, p > 0.05).

Overall significant differences between spoil tips and peripheral environments were also achieved in similar comparisons on each vegetation cover separately. Indeed, differences between spoil tips and peripheral environments on each vegetation cover were observed for grain size classes (granulometry) (Table 1), as well as for parameters impacted by activity in the soil (meaning nitrogen, organic matter of C/N ratio), when vegetation was not yet developed. However, no differences were observed in shrub and tree covers between spoil tips and peripheral environments (Table 1, for shrub and tree covers in nitrogen, organic matter and C/N measures).

### Collembola communities

#### Spoil Tips vs. Peripheral Environments

**Figure 2:**
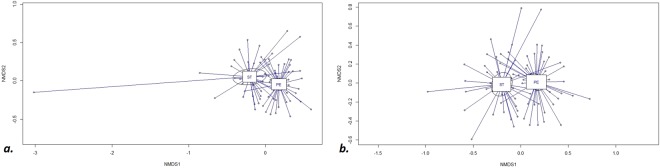
Non Metric data Scaling. Projection of station in the plane of the first two factorial axes. (**a**) with the representation of the station ST12B ADONIS r^2^ = 0.062 p < 0.005. (**b**) without ST12B. r^2^ = 0.059 p < 0.005. ST = Spoil tip stations. PE = surrounding stations. The ellipses corresponded to 95% CI.

A strong significant difference of collembola communities was observed between spoil tips and in peripheral environments (Fig. 2a ADONIS r^2^ = 0.048, p ≤ 0.01). The corresponding stress test result was 0.238 (<0.3), suggesting that the graphical representation of Fig. 2a was close to reality. The difference was always observed except for the very extreme point corresponding to the ST12B station, only represented by one Collembola (Fig. 2b). From now on, analysis will be performed without the ST12B station.

**Figure 3:**
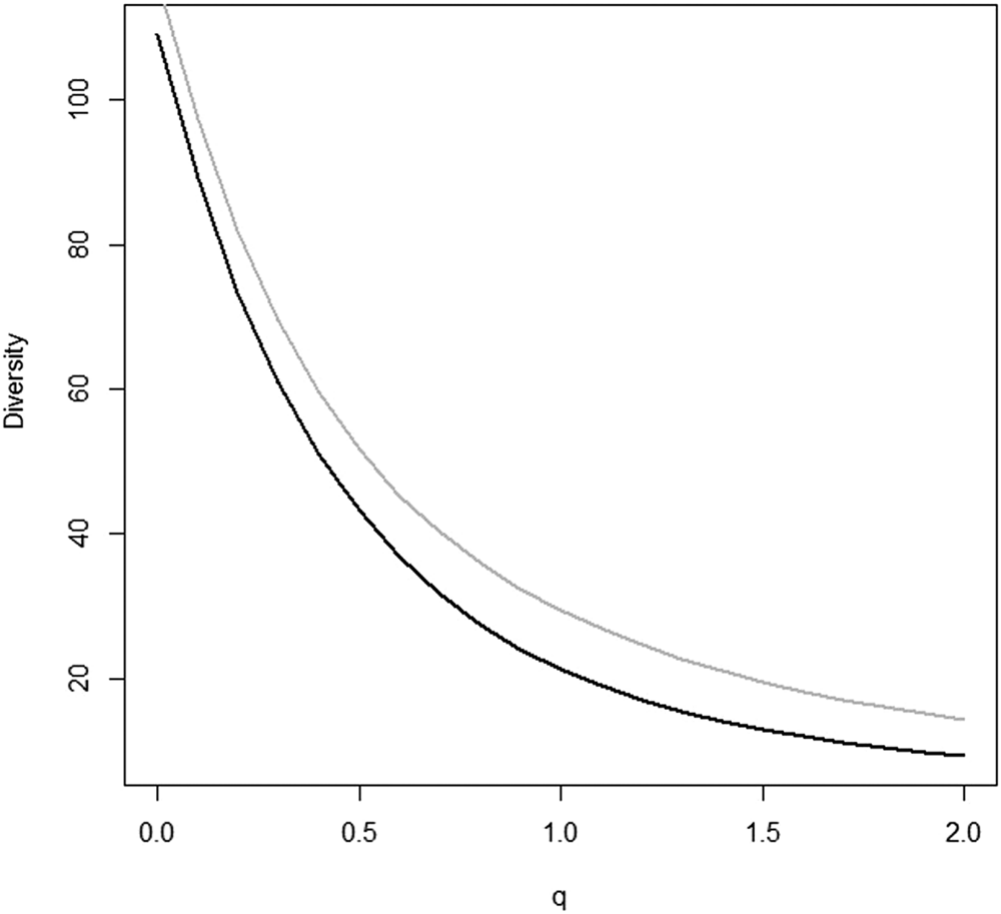
Renyi biodiversity profile of spoil tip springtail assemblage (black line) vs biodiversity profile of native environment (grey line), according to the Hill’s number (see materials and methods for more explanation). The Renyi biodiversity profiles showed Hill’s number indices of the communities ^0^*D* = *S* (species richness), ^1^*D* = *e*^*H*^ the Shannon index exponential, and ${}^{2}D=\frac{1}{1-E}$ the inverse of the Gini-Simpson concentration indice.

Diversity profiles of collembola assemblages from peripheral environments and spoil tips were distinct as they did not intersect (Fig. 3). Moreover, collembola assemblages from the peripheral environments were more diverse than in spoil tips (Fig. 3, grey line). Finally, the shape of the curves showed that both communities were characterized by a few species dominance and a large number of rare species. Indeed, only six species in peripheral environments and four species in spoil tips represented more than 50% of all individuals. *Parisotoma notabilis*, *Lepidocyrtus lanuginosus*, *Isotoma viridis* and *Folsomia quadrioculata* were four of the five more abundant species in both locations. In all of our samples, *Hemisotoma thermophila* was the third most abundant species overall but was mostly observed on spoil tips (96.4% of *H*. *thermophila* individuals came from spoil tips).

**Figure 4:**
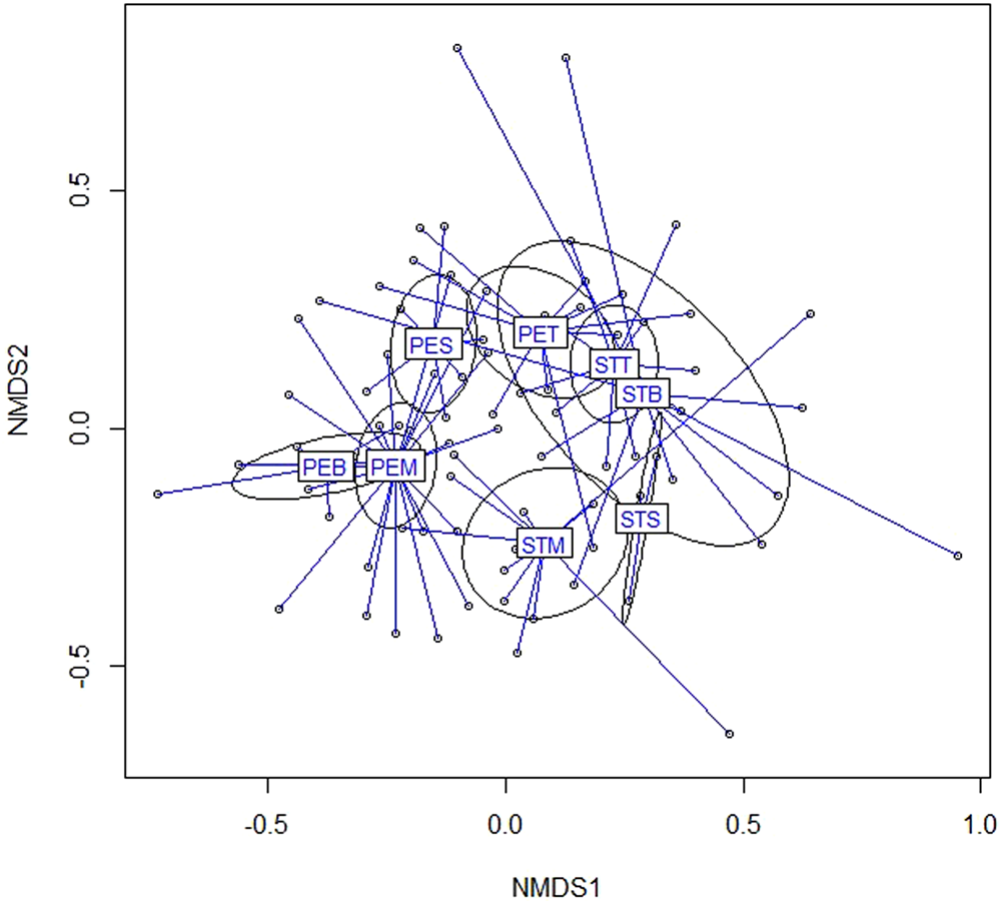
Non Metric data Scaling. Projection of stations in the plane of the first two factorial axes. STB = Bare Spoil tip stations. STM = Meadow Spoil Tip stations STS = Shrub Spoil Tip stations, STT = Forest Spoil Tip stations, PEB = Bare surrounding stations, PEM = Meadow surrounding stations, PES = Shrub surrounding stations, PET = forest surrounding stations. ADONIS r^2^ = 0.21 p < 0.005. The ellipses mean 95% CI.

**Figure 5:**
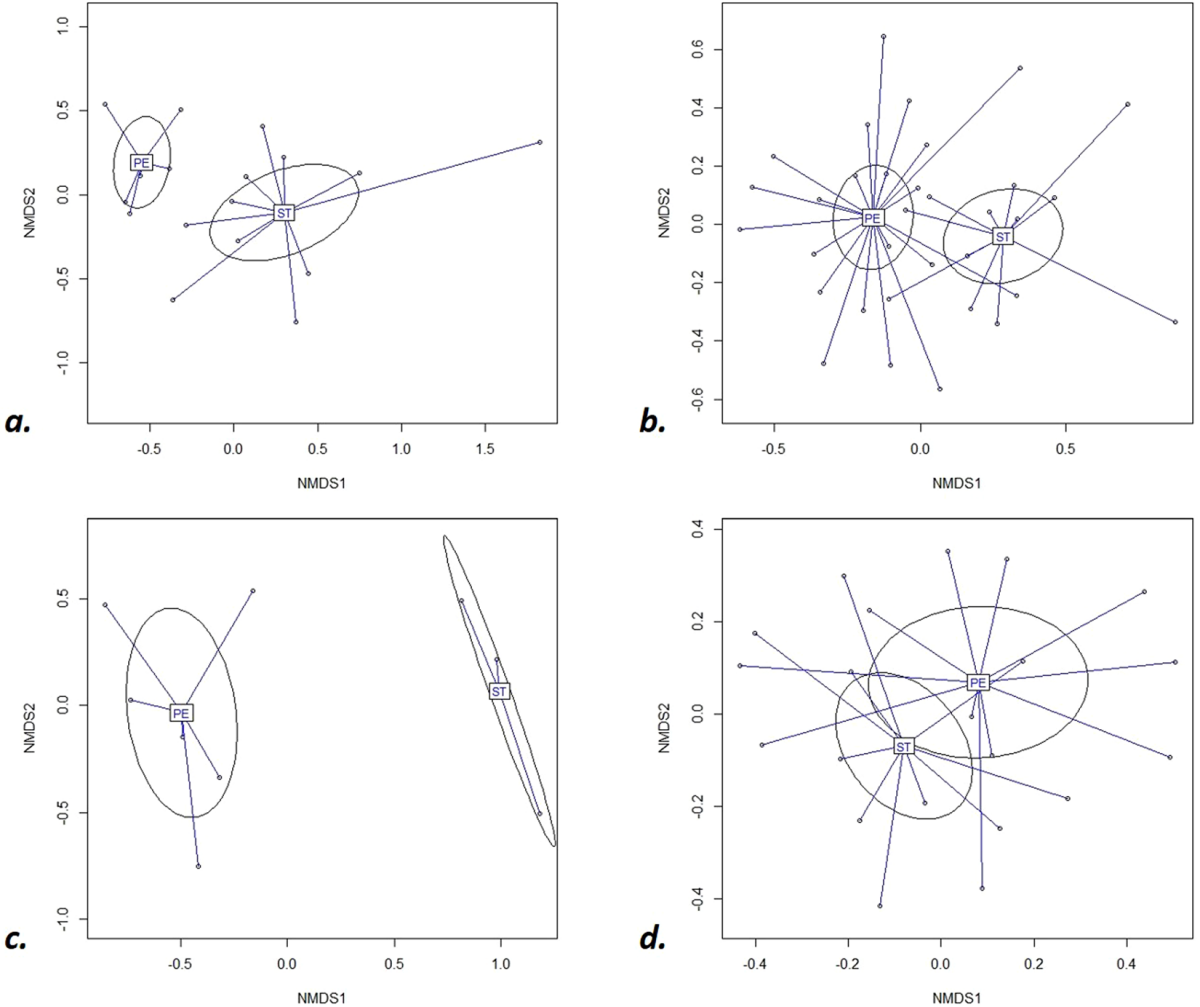
Comparison of collembola assemblages between spoil tips (ST) and surrounding (PE) for (**a**) bare stations R^2^ = 0.18 p < 0.005, (**b**) meadow stations ADONIS R^2^ = 0.07 p < 0.005, (**c**) shrub stations ADONIS R^2^ = 0.28 p < 0.05, (**d**) forest stations ADONIS R^2^ = 0.05 p = 0.47. The ellipses mean 95% CI.

Differences in collembola communities between spoil tips and peripheral environments also manifested according to vegetation covers (Fig. 4). Multifactorial analysis demonstrated distinct communities along the first axe, caused by a true segregation between spoil tips and peripheral environments communities. Those differences were also visualized by analysis of vegetation covers omitting tree stations (Fig. 5). This difference was statistically confirmed in bare soils, meadows and shrub (Fig. 5, ADONIS, p < 0.005 for bare, meadow stations and p < 0.05 and p > 0.05 for shrub and tree stations, respectively).

Nine “pioneer” species were highly specific of spoil tips (Table 2). Among them, *Brachystomella parvula*, *Folsomides parvulus*, *Paratullbergia callipygos and pseudosinella cf*. *terricola* were the most abundant. However, none of them exceeded 5% of total individuals caught (Table 2). On the contrary, five “stenoecious” species were exclusively found in peripheral environments (Table 2). None of them exceeded 2% of total individuals caught (Table 2). Those species, e.g. *Dicyrtoma fusca*, *Isotomurus palustris*, *Pseudosinella immaculata*, *Stenaphorura denisi* and *Stenaphorura quadrispina*, were never observed on spoil tips along the study.

**Table 2:** Soil preference and vegetation-cover preference for principal springtail species inventoried in the coal mining field in north of France. N = Number of individuals Soil preference: PE = Peripheral Environment, ST = Spoil Tip.

Species name	N=	Substrata preference	Species qualif.	Dunger & al. 2004	Vegetation-cover preference	Species qualif.	Auclerc & al. 2009	Ponge *et al*. 2005
*Allacma fusca*	44	PE	Opportunist	NA	T*	Forest-exclusive	Forest specialist	Woodland
*Bourletiella hortensis*	25	ST	Colonizing	Pioneer	T	Forest prefering		
*Brachystomella parvula*	149	ST**	Pioneer		M	Meadow exclusiv		Agri. land
*Ceratophysella denticulata*	152	ST	Colonizing	Stenoecious	M	Meadow elective	Meadow-preferring	Agri. land
*Ceratophysella gibbosa*	20	ST	Colonizing		T	Forest elective		
*Proisotomodes bipunctatus*	24	PE	Opportunist		B	Bare elective		
*Hemisotoma thermophila*	692	ST	Colonizing		B	Bare preferring		
*Cyphoderus albinus*	15	ST	Colonizing		M*	Meadow-exclusive		Agri. land
*Desoria trispinata*	58	PE	Opportunist		T	Forest elective		
*Desoria violacea*	51	PE	Opportunist	Pioneer	M	Meadow elective		
*Deuterosminthurus pallipes*	11	IND	indifferent		S	Shrub preferring		
*Dicyrtoma fusca*	128	PE*	Stenoecious	Euryece	S**	Shrub-exclusive		Agri. land
*Dicyrtomina minuta*	17	ST	Colonizing	Stenoecious	BMS	Indifferent	Forest specialist	Agri. land
*Entomobrya corticalis*	22	ST	Colonizing	NA	T	Forest-exclusive		
*Entomobrya lanuginosa*	40	PE	Opportunist		S	Shrub preferring		
*Entomobrya multifasciata*	152	PE	Opportunist	Pioneer	M	Meadow preferring	Forest specialist	Agri. land
*Entomobrya nicoletti*	39	ST	Colonizing		S	Shrub preferring		
*Folsomia spinosa*	23	PE	Opportunist		T	Forest elective		
*Folsomia candida*	19	PE	Opportunist	Stenoecious	B	Bare preferring		Agri. land
*Folsomia fimetaria*	32	ST	Colonizing		M	Meadow elective		Agri. land
*Folsomia manolachei*	178	PE	Opportunist		MST	Indifferent		
*Folsomia quadrioculata*	882	ST	Colonizing	Stenoecious	T	Forest elective	Forest-preferring	Woodland
*Folsomides parvulus*	241	ST***	Pioneer		S	Shrub preferring		
*Friesea cf*. *handschini*	30	ST**	Pioneer		S*	Shrub-exclusive		
*Friesea mirabilis*	13	ST	Colonizing	Pioneer	T	Forest prefering		Woodland
*Friesea truncata*	75	PE	Opportunist		T**	Forest-exclusive	Forest specialist	Woodland
*Friesea villanuevai*	40	ST	Colonizing		M	Meadow elective		
*Heteromurus major*	52	ST	Colonizing		M	Meadow elective		
*Isotoma anglicana*	69	PE	Opportunist	NA	M**	Meadow-exclusive	Meadow-preferring	
*Isotoma viridis*	544	PE	Opportunist	Pioneer	M**	Meadow-exclusive		Agri. land
*Isotomiella minor*	106	ST	Colonizing	Euryece	T	Forest preferring	Forest specialist	Woodland
*Isotomodes productus*	51	ST	Colonizing	Pioneer	M	Meadow elective		Woodland
*Isotomurus palustris*	112	PE*	Stenoecious	Pioneer	M	Meadow elective		Agri. land
*Isotomurus prasinus*	127	PE	Opportunist		M	Meadow elective		
*Kalaphorura burmeisteri*	27	ST	Colonizing		T	Forest-exclusive		Agri. land
*Lepidocyrtus curvicollis*	42	ST*	Pioneer		T	Forest elective		
*Lepidocyrtus cyaneus*	319	PE	Opportunist		M*	Meadow-exclusive	Meadow specialist	Agri. land
*Lepidocyrtus lanuginosus*	590	ST	Colonizing	Pioneer	T	Forest preferring	Generalist	Woodland
*Lepidocyrtus lignorum*	60	ST	Colonizing	NA	B	Bare preferring	Generalist	Agri. land
*Lepidocyrtus violaceus*	35	PE	Opportunist	Pioneer	T	Forest prefering		
*Mesaphorura florae*	14	PE	Opportunist	Pioneer	S	Shrub preferring	Meadow specialist	
*Mesaphorura macrochaeta*	213	ST	Colonizing	Pioneer	T***	Forest-exclusive	Generalist	Woodland
*Mesaphorura sylvatica*	31	ST**	Pioneer	NA	T	Forest elective		
*Metaphorura affinis*	109	ST	Colonizing	Pioneer	B	Bare elective		
*Monobella grassei*	33	ST***	Pioneer		T	Forest prefering		
*Neanura muscorum*	68	PE	Opportunist	Pioneer	S*	Shrub-exclusive	Forest specialist	Woodland
*Neonaphorura duboscqi*	23	ST	Colonizing		T	Forest-exclusive		
*Oconpodura crassicornus*	22	ST	Colonizing		S	Shrub preferring		Woodland
*Orchesella cincta*	46	ST*	Pioneer		T	Forest prefering	Forest specialist	Woodland
*Orchesella villosa*	20	IND	indifferent	NA	M	Meadow preferring		Agri. land
*Paratullbergia callipygos*	261	ST**	Pioneer	Euryece	T*	Forest-exclusive	Forest specialist	Woodland
*Parisotoma notabilis*	2804	ST	Colonizing	Pioneer	T**	Forest-exclusive	Meadow-preferring	Agri. land
*Pogonognathellus longicornis*	31	PE	Opportunist	Euryece	S	Shrub preferring		
*Proisotoma minuta*	67	PE	Opportunist	Pioneer	M	Meadow elective		
*Protaphorura armata*	275	PE	Opportunist	Pioneer	T	Forest preferring		Agri. land
*Protaphorura aurantiaca*	59	IND	Indifferent		T	Forest-exclusive	Meadow-preferring	
*Protaphorura octopunctata*	48	ST	Colonizing		T	Forest-exclusive		
*Pseudisotoma sensibilis*	25	PE	Opportunist		T	Forest-exclusive		Woodland
*Pseudosinella alba*	348	PE	Opportunist	Euryece	S*	Shrub exclusive	Meadow-preferring	Agri. land
*Pseudosinella immaculata*	39	PE**	Stenoecious		S	Shrub-preferring		
*Pseudosinella cf*. *terricola*	286	ST***	Pioneer		B***	Bare-exclusive		
*Sminthurides malmgreni*	24	ST	Colonizing		BMST	Indifferent		
*Sminthurinus aureus*	31	PE	Opportunist	Euryece	T*	Forest exclusive	Meadow-preferring	Agri. land
*Sminthurinus elegans*	289	ST	Colonizing		S	Shrub-preferring		
*Sminthurinus niger*	56	PE	Opportunist	NA	T	Forest preferring		Agri. land
*Sminthurus viridis*	15	PE	Opportunist		S*	Shrub exclusive	Meadow-specialist	Agri. land
*Sphaeridia pumilis*	115	PE	Opportunist	Pioneer	S	Shrub preferring	Meadow-specialist	Agri. land
*Stenaphorura denisi*	22	PE**	Stenoecious	Stenoecious	S	Shrub preferring	Meadow-specialist	Agri. land
*Stenaphorura quadrispina*	43	PE**	Stenoecious	Euryece	M	Meadow preferring		Agri. land
*Supraphorura furcifera*	25	PE	Opportunist		T	Forest exclusive		
*Pogonognathellus flavescens*	13	ST	Colonizing	Pioneer	T*	Forest-exclusive		Woodland
*Tomocerina minuta*	70	PE	Opportunist		S	Shrub preferring		
*Tomocerus vulgaris*	16	PE	Opportunist	Pioneer	T	Forest elective		
*Xenylla grisea*	22	ST	Colonizing		T	Forest elective	Forest-specialist	Woodland
*Xenylla tullbergi*	73	IND	indifferent		T*	Forest exclusive	Forest-specialist	Woodland

Vegetation-cover preference: B = Bare, M = Meadow, S = Shrub, T = Tree.

*Significance level ≤ 0.05, **significance level ≤ 0.01, ***significance level ≤ 0.001.

The substrata preference of those four species was unresolvable (cf. IND in Table 2), partly because none of them had a high number of individuals.

The other 57 species were either opportunist (with slight preference for peripheral environments) or colonizing (with slight preference for spoil tips).

#### Difference between vegetation covers (Bare vs. Meadow vs. Shrub vs. Tree stations)

**Figure 6:**
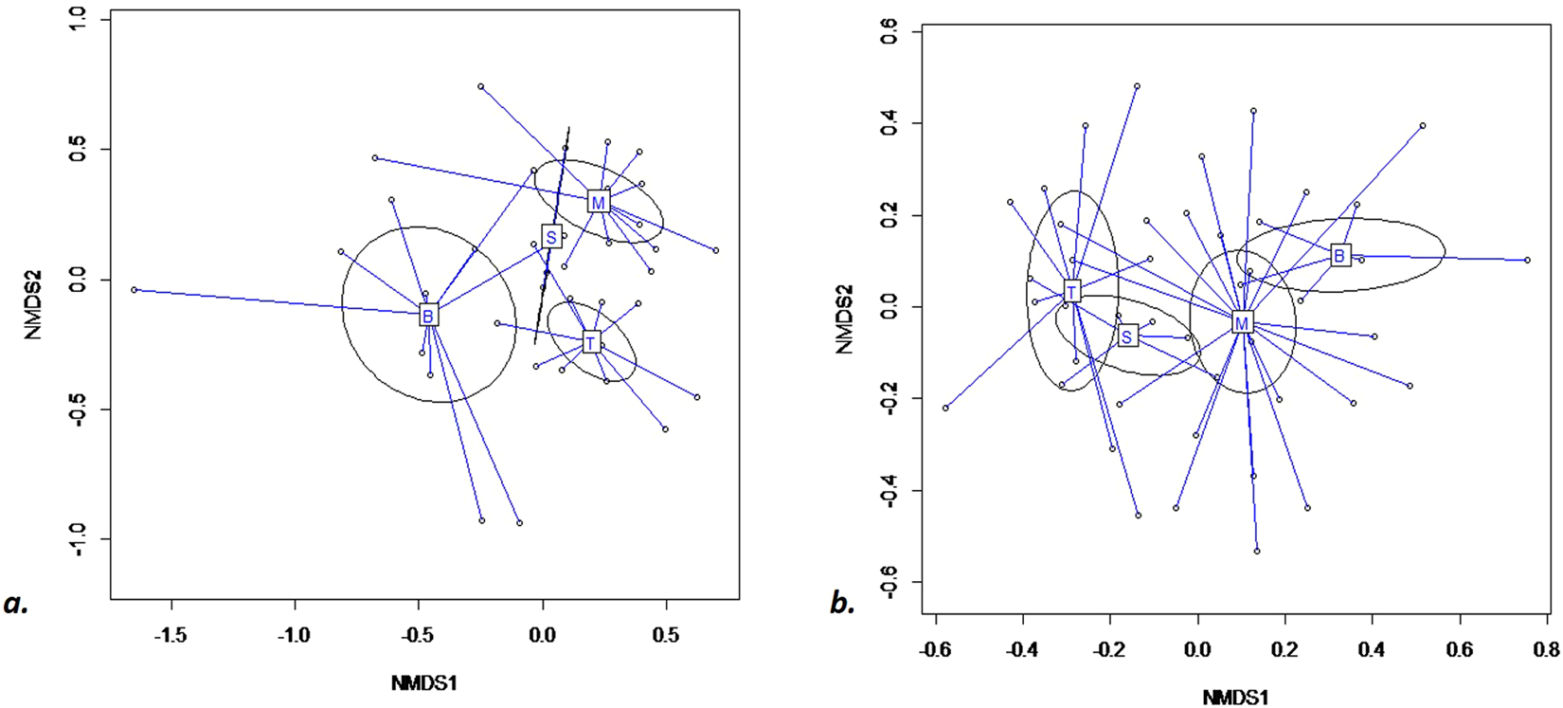
Comparison of collembola assemblages per vegetation cover. (**a**) in spoil tips ADONIS R^2^ = 0.18 p < 0.005 (**b**) in surrounding environment ADONIS R^2^ = 0.15 p < 0.005. B: bare stations, M: meadow stations, S: shrub stations, T: forest stations. The ellipses mean 95% CI.

The differences in collembola assemblages according to vegetation cover were significant in both substrate conditions (Fig. 6a: for spoil tip and Fig. 6b for peripheral environments). Indeed, an overall difference between collembola communities in vegetation cover stages was previously observed (Fig. 4). Moreover, successive community stage along the vegetation gradient was also obvious along the second axe of multifactorial analysis (Fig. 6a,b), even more in peripheral environment of spoil tips (Fig. 6b).

**Figure 7:**
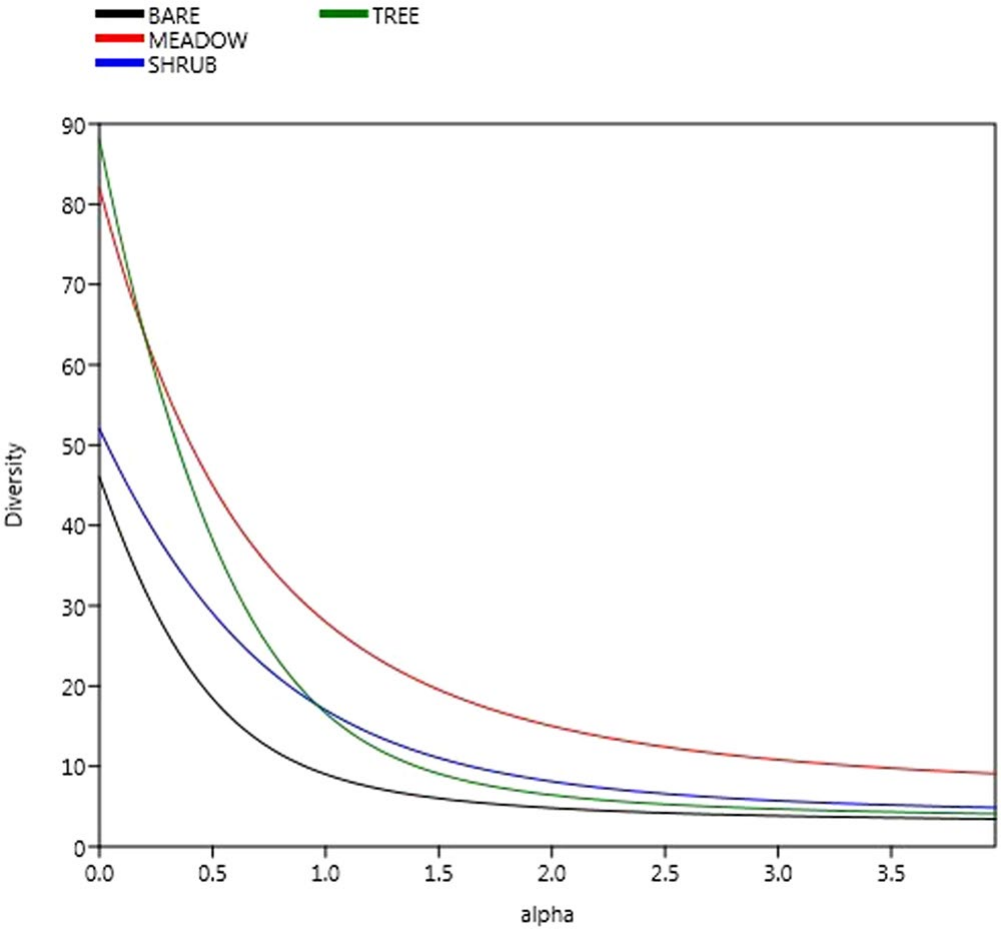
Diversity profiles of collembola communities (Hill’s number in relation to the weighing parameter q, “alpha” here) carried out on different vegetation covers whatever on spoil tip or peripheral environment. The Renyi biodiversity profiles showed Hill’s number indices of the communities ^0^*D* = *S* (species richness), ^1^*D* = *e*^*H*^ the Shannon index exponential, and ${}^{2}D=\frac{1}{1-E}$ the inverse of the Gini-Simpson concentration indice.

Diversity profiles performed on vegetation covers (Fig. 7) demonstrated that tree stations had higher number of species, represented mostly by rare species. Overall, meadow stations were the most diverse stations. Finally, bare stations were the least diverse stations since the diversity profile was distinctly under all the other curves.

Tree stations contained the most diversified collembola assemblage with 32 species among the 75 species studied. Since half of them (15) were found only in tree stations, they were classified as forest-exclusive (Table 2). *Parisotoma notabilis* was the most abundant species found in this category (more than 25% of all individuals).

Shrubs and meadows stations contained 17 species together. Five species were only found in shrub cover. Among them, *Pseudosinella alba* (3% of individuals) and *Dicyrtoma fusca* (1.1% of individuals) were the most abundant. *Isotoma viridis* (5% of individuals) was unique to meadow stations (Table 2).

Finally, with only six species, bare stations seemed to be the least favorable biotope for Collembola. *Pseudosinella cf*. *terricola* (2.6% of individuals) was the most abundant species, exclusive to bare stations. Although found in other stations, *Hemisotoma thermophila* demonstrated a preference for bare stations (78% of *H*. *thermophila* individuals caught (N = 692) were in bare stations).

## Discussion

In this study, we demonstrated that both substrata and vegetation cover strongly impact the collembola assemblages. Moreover, we established the following three points: (i) differences in substrata is responsible for the difference in Collemba species assemblages between spoil tips and peripheral environments; (ii) a difference between substrates is also observed for each vegetation cover studied (bare, meadow and shrub stations) but not for tree cover; (iii) significant differences are observed between successive vegetation covers on spoil tips as well as on peripheral environments, regardless of the substrate.

### Overall Difference Between Spoil Tips And Peripheral Environments

The comparison between spoil tips and peripheral environments highlighted specificities in each sites. First, physico-chemical analysis are significantly different. Such results are consistent with previsous studies pointing out differences in soil texture, organic matter, nitrogen content^103,104^ in colliery waste heaps in Northern France^10,105^. Similarly, collembola assemblages in this study on spoil tips are also different from those observed in surrounding environments. Hence, in Northern France, spoil tips are islands of schist substrate in the middle of regional, interconnected landscape represented by cities, field or woodland on clay/silt mixed substrate. In this context, migration abilities of species and their adaptation for such a rough environment are immediatly questionned. Indeed, spoil tips are known to be warmer and drier than peripheral environment^104^. Consequently, these rough conditions might play a role of emvironemental filter^103,106^ and result in a functional traits variation of regional species^10,107^. However, those conditions can also be factors of adaptation for other species^108–110^. This is particularly relevant for groups of species like Collembola for which the most important environmental factors for their growth and their development are temperature, humidity and food.

Moreover, beyond functional traits restoration with consequences on functional ecology, it is critical to maintain, at a regional scale, a strong specificity at the species level^106^. A previous study^10^ based on functional-trait values showed value stability across stations. However, our analysis based on species diversity (identity and abundance), strong variations in species composition are observed across stations, mainly between spoil tips and peripheral environment. Such differences in a pool of species are not observed in a functional trait approach. This validates the importance of studying both species diversity and functional biodiversity in a complementary manner.

### Differences Between Spoil Tips And Peripheral Environments According To Vegetation Covers (Except Tree Cover)

Differences in species community between spoil tips and their peripheral environments are also noticed comparing vegetation covers, at the exception of the forest.

#### Bare soils

In the peripheral environments, bare soils show different species than bare soil from spoil tips. This result confirms the large impact of soil substrate on the collembola species composition. Indeed, without any vegetation, substrate is the only factor changing species distribution in bare soils. However, one surprising observation is the presence of relatively abundant Collembola on such a bare soil biotope. Indeed, bare soils are considered poor in resources and instable due to the lack of plant roots and/or slope in spoil tips. While it is the less diverse biotope, some species like *Hemisotoma thermophila* are found on bare soils in high number. Despite the absence of any vegetation and decomposition products derived from photosynthesis, Collembola might find other food resources such as bacteria or fungi. Indeed, a relationship between fungi and Collembola has been previously mentioned, and may help both populations self-maintain despite the lack of abundant vegetation^111^. Beyond subsistence, spoil tips have a hard microclimate ranging from black color to great slope (for conical spoil tips), which increase temperatures and dryness. The outcome is a variation of collembola assemblages that were better adapted to bare soils on spoil tips than peripheral environment. In addition, colonization might be also enhanced by an absence of predation^112^. In fact, spoil tips inhospitable features also impact all species groups including predators^105^. In rough conditions (here, bare stations without vegetation), collembola assemblages are a melting pot of opportunistic species and rare species, part of the “pioneer” group sensu Dunger *et al*.^97^. For example, local pioneers are observed in high number in skeletal soil such as *Folsomides parvulus* and *Metaphorura affinis*. However, some rare species are also found. For example, *Pseudosinella cf*. *terricola*, is exclusive to bare soils on spoil tips and has been defined as a new regional taxa for this study. This species may be either undescribed coming from another country or a particular population of *Pseudosinella sexoculata* Schoett 1902 with only five ocelli. Currently, the second hypothesis is preferred since the species was previously identified on British spoil tips^113^. However, the exceptional features of spoil tip, compared with their surrounding environment, might also promote exotic species. Indeed, this has been previously demonstrated for many fauna and/or flora taxa^105^ and Collembola is not an exception. For example, *Monobella grassei* or *Friesea villanuevai* have an unknown colonizing path from their original southern regions (Southern France for *M*. *grassei*^114^ and Spain for *F*. *villanuevai*^115^). These species find sanctuary in the warm stations such as spoil tips. Moreover, even if these species are not exclusive to bare soil in this study, their presences show that the lack of vegetation on technosoil presents an opportunity with environmental tolerance. However, with vegetation growth, other species may dominate them and counteract the maintenance of those populations confined to bare soils.

#### Meadow and shrub covers

In meadow and shrub covers, collembola assemblage differences between spoil tips and peripheral environments could be first induced by the previously described difference in bare soils. But, it may also be linked to vegetation growth according to substrate difference and/or water and nutrients availability^116–118^. Finally, differences might also vary depending on landscape characteristics. Indeed, two succession vegetation paths as a function of a ruderal or non-ruderal definition of landscapes has been previously considered^119^. In the spoil tips of Northern France, reclamation is based on slight human intervention allowing spontaneous successive vegetation, which lead to increased habitat and species diversity^120^ in^121^, on the contrary to surrounding environments regularly managed by humans. Moreover, modification of soil environment (organic matter rate, aggregate formation…) also affects composition of soil biota communities^122^ and the reverse is also true: soil fauna impacts soil formation^44,123,124^. Thus, before climax, species distribution occurs through various pathways and subsequent species assemblages, which is not so precisely observed in trait modalities^10^.

#### Tree covers

With time and vegetation growth, vegetation cover thickness increases and becomes more complex. Similarly, collembola communities homogenize across the region. As a result, no differences are observed in samples from forest in spoil tips as well as surrounding environments. Despite substrate differences, identical forest soil communities are noticed in both locations. This result differs from previous study, notably in the Berzdorf mine forest site investigation^97^. One explanation may be that different vegetation dynamics are observed. Indeed, in the Berzdorf study, human action was strong, notably by afforesting part of spoil tips. On the contrary, Northern France policy promotes natural growth dynamic. Nevertheless, further investigations are necessary to explain such differences, e.g. the analysis of the two systems, the first afforested and managed by humans and the second focused on natural dynamics.

### The Use Of Species To Define Environment

#### Difference between different vegetation covers

Many studies have already demonstrated than successive vegetation covers present different communities of Collembola^125^. This was confirmed by our results and echoed back to our previous analysis by trait values^10^. Because of the depth origin and strong mineralization process, initial bare soils from spoil tips present limited organic resources for soil fauna^103^, strongly affecting the local species diversity. With the natural chrono-sequence in vegetation on abandoned spoil tips also observed in other industrial sites^42^, part of successive soil fauna assemblage disappears and is replaced by vegetation^43^. However, clear collembola assemblages correlating with specific vegetation covers is not observed. Indeed, species could be classified according to their affinity for one, two, three or four stages of vegetation but never exclusive to one of them. Moreover, according to Dunger *et al*.^97^, no stable association of collembola species is observed on vegetation covers on spoil tips or peripheral environments. Despite these constraints, the use of the $\,indval\,\,$index method^95^ allows the definition of specificity level and the identification of species groups within bio-indication interest. Accordingly, collembola species are categorized as exclusive, elective, preferred or indifferent species. The first two, and to a lesser degree the third one, can be used to define the adaptation to a distinct biotope. Beyond their functional traits, many studies converge towards the definition of species to specifically cover vegetation. Indeed, our results corroborate many other surveys^46,47,83^. Hence, we confirmed that *Allacma fusca*, *Folsomia quadrioculata*, *Friesea mirabilis*, *Friesea truncata*, *Isotomiella minor*, *Orchesella cincta*, *Paratullbergia callipygos*, *Pseudisotoma sensibilis*, *Pogonognathellus flavescens*, *Xenylla grisea* and *Xenylla tullbergi* are forest species. We also confirmed that the following species are specific of meadow (or agriculture land): *Brachystomella parvula*, *Ceratophysella denticulata*, *Cyphoderus albinus* (Myrmecophile species), *Isotoma anglicana*, *Isotoma viridis*, *Isotomurus palustris*, *Lepidocyrtus cyaneus*, *Pseudosinella alba*, *Sminthurus viridis*, *Sphaeridia pumilis*, *Stenaphorura denisi* and *Stenaphorura quadrispina*.

#### Differences between spoil tips and peripheral environments

Some species allow the distinction between substrates and thus, make the separation between spoil tips and surrounding environments. Consequently, the use of collembola species as a surrogate of physicochemical measures is becoming relevant and might improve the analysis performed with functional traits^10^. This study is in line with soil indicators development based on collembola diversification^126^, notably using ecomorphology^57,127^ or other approaches often complex but more complete^128^. Mostly, species caught only in spoil tips are considered as an indicator of the anthropogenic environment linked with technosoil. In our study however, *Brachystomella parvula* is a spoil tips-specific species generally found in urban systems^14,65^. Therefore, some species might indicate disturbance rather than a real environment typology. For example, *B*. *parvula* is typical of lawns^14^, regularly managed by humans. Species in the pioneer group^97^ have the ability to colonize (generalists, surface active or parthenogenesis^129,130^), which explains the colonization of abiotic environments despite poor organic matter on the ground. This also enlightens why they are more often found in an urban context (or more generally in anthropogenic environments characterized by certain instability).

More interestingly, the presence of particular indicator species allows us to better define the environment and sometimes to go back over the site history. For example, *Folsomides parvulus*, specific to spoil tips, is observed in one peripheral station. After further investigation, we discovered that this particular environment is an ancient railway on a schist roadbed, similar to spoil tip stations. Consequently, it is also possible to retrace history thanks to some collembola species^56^. Similarly, a large population of *Hemisotoma thermophila* in the bare station of spoil tips T108 of Ostricourt raises questions as this species is usually enhanced by organic matter^74^. Site analysis further reveals that this bare station is an ancient afforested station cleared 2 years before our study. As a result, collembola species represents a soil memory indicator in species diversity analysis (mainly species identity of but also their respective abundance).

### Limits Of The Use Of Species As Indicators

Despite the similarity in species specificities in our study and others, few species retain our attention and more in-depth studies are needed to clarify their status. For example, *Dicyrtomina minuta* and *Entomobrya multifasciata* are a forest-specific species by Auclerc *et al*.^46^ whereas in our hand, they are meadows species such as observed by Ponge *et al*.^47^. Again, generalist species *Lepidocyrtus lanuginosus* and *Mesaphorura macrochaeta* in Auclerc *et al*.^46^ are described as two forest-specific species in our study as well as Ponge *et al*.^47^. However, for *Lepidocyrtus lanuginosus*, the existence of cryptic species^131^ makes the analyses difficult. Indeed, Auclerc *et al*.^46^ and Ponge *et al*.^47^ may have, just like us, worked on two different lineages with different habitat specificities.

*Dicyrtoma fusca*, *Sminthurinus aureus* and *Neanura muscorum* are shrub species in our survey. However, *D*. *fusca* and *S*. *aureus* are found in meadow and/or forest litter in other studies^132^. *N*. *muscorum* has been consistently a forest species in the literature, under rotting wood^87^ or in the soil^132^. However the difficulty with shrub station such as defined here is the fact that bushes are barely taken into account independent of trees.

Our study also questions the limits of the *indval* index. In our hand, individuals of *P*. *notabilis* are found in nearly all samples while other studies consider it as ubiquist^133^. Nevertheless, *indval* index classifies *P*. *notabilis* as a forest-specific species. In this case, abundant litter might improve the reproduction of specific species. Thus, $indval$ results are skewed by strong differences in abundances between different vegetation covers (and consequently litter quantity) rather than reflecting real specificity.

*Protaphorura aurantiaca* is the only species for which preference interpretation strongly diverges from our data. In our hand, *P*. *aurantiaca is a* forest-specific species while other studies define it as meadows-specific^46,47^. However, one publication also shows a forest preference for this species^134^. Despite a distinct chaetotaxy for discrimination inside the complex genus *Protaphorura “armata”*^135^, we suggest that this taxon requires a taxonomic revision^136^. Such revision could result in the novel definition of species, which then would correspond to different ecological preferences.

### Beyond The Functional Traits, Spoil Tips As The Refuge Of Particular Species

Previous study showed that, from the traits value perspective, an equilibrium in spoil tips is noticed^10^. However, complementary analysis using the species approach highlights the importance of such an environment for regional biodiversity^105^ and conservation biology^7,137^. In this study, critical observations of collembola species are made and new species discovery on a local scale have occurred. For example, rare species such as *Pseudosinella sexoculata* Schoett 1902^113^, exotic thermophile species like *Proctostephanus madeirensis* da Gama 1959^113^ or new regional species such as *Mesaphorura atlantica* Rusek 1979^79,113^ are unique discovery in the regional landscape. Moreover, in our studies, three thermophiles species are new for Northern France: *Heteromurus major*, *Monobella grassei* and *Friesea villanuevai*.

Finally, in addition to previous one^10^, we also confirm the benefit of pursuing biodiversity analysis at both the level of functional traits and of species determination to draw a complete picture of the ecological process. These complementary approaches will surely enhance the management of unique sites, such as spoil tips, having to deal with simultaneous issues in restoration ecology and conservation biology.

## Electronic supplementary material

Supplementary Information
