# Supplementary material for: Differences in collembola species assemblages (Arthropoda) between spoil tips and surrounding environments are dependent on vegetation development
Source: Sci Rep. 2018 Dec 24;8:18067. doi: 10.1038/s41598-018-36315-1 (PMC6305484; doi:10.1038/s41598-018-36315-1)
Supplement: Supplementary file 1 — Supplementary Information [file 41598_2018_36315_MOESM1_ESM.pdf]

Differences in collembola species assemblages (Arthropoda) between spoil tips and surrounding environments are dependent on vegetation development.

Vanhée Benoit[1] & Devigne Cédric[1*]

1. Equipe Ecologie & Biodiversité, Faculté de Gestion Economie & Sciences, université Catholique de Lille – 60 Bvd Vauban – 59016 Lille cedex – France

*Corresponding author: cedric.devigne@univ-catholille.fr

Supplementary information

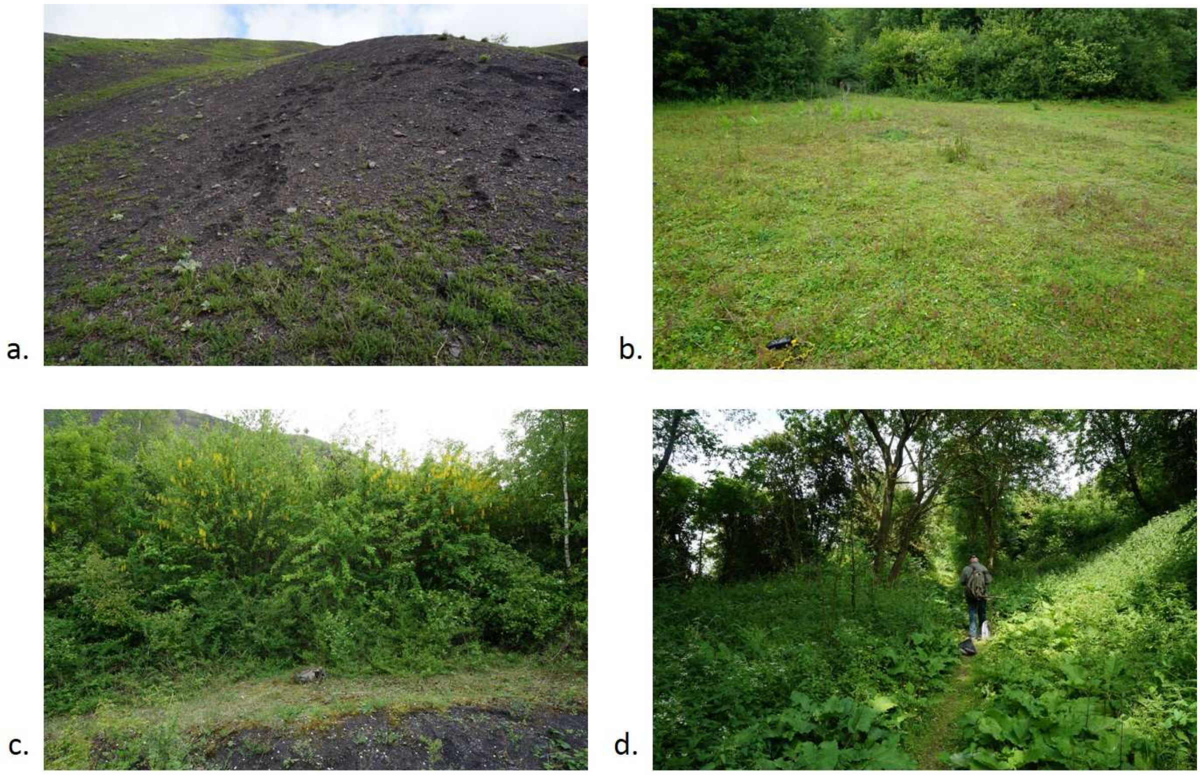

Figure S1. Pictures on the different vegetation covers studied. *a.* Bare station, *b.* Meadow station, *c.* Shrub station and *d.* Tree station

Table S1: sites of springtails community analysis, with the number of stations, the vegetal cover, the substrata and the locality. ST = Spoil Tip, PE = Peripheral Environment, St = Numbre of stations (5 samples carried out on each of these stations).

Name	St.	Vegetation cover	substrata	locality
ST2B	2	Bare	ST	Ruitz
ST2M	1	Meadow	ST	Ruitz
PE2M	3	Meadow	PE	Ruitz
ST2S	1	Shrub	ST	Ruitz
PE2T	1	Tree	PE	Ruitz
ST7M	2	Meadow	ST	Ruitz
ST7T	1	Tree	ST	Ruitz
ST9B	1	Bare	ST	Haillicourt
ST9M	2	Meadow	ST	Haillicourt
ST9S	1	Shrub	ST	Haillicourt
ST9T	1	Tree	ST	Haillicourt
PE9M	1	Meadow	PE	Haillicourt
PE9S	1	Shrub	PE	Haillicourt
PE9T	1	Tree	PE	Haillicourt
ST11B	1	Bare	ST	Bruay B.
PE11M	1	Meadow	PE	Bruay B.
PE11T	3	Tree	PE	Bruay B.
ST12B	1	Bare	ST	Bruay B.
ST12M	1	Meadow	ST	Bruay B.
ST12T	2	Tree	ST	Bruay B.
PE12M	3	Meadow	PE	Bruay B.
PE12T	1	Tree	PE	Bruay B.
ST11T	3	Tree	ST	Bruay B.
ST56B	1	Bare	ST	Verquin
ST56M	1	Meadow	ST	Verquin
ST93B	1	Bare	ST	Harnes
ST93M	1	Meadow	ST	Harnes
ST93T	1	Tree	ST	Harnes
PE93T	1	Tree	PE	Harnes
ST98B	1	Bare	ST	Estevelles
ST98M	1	Meadow	ST	Estevelles
ST98S	1	Shrub	ST	Estevelles
ST98T	2	Tree	ST	Estevelles
PE98M	2	Meadow	PE	Estevelles
PE98T	1	Tree	PE	Estevelles
ST108B	1	Bare	ST	Ostricourt
ST108T	1	Tree	ST	Ostricourt
PE108T	1	Tree	PE	Ostricourt
ST115B	1	Bare	ST	Libercourt
ST115M	2	Meadow	ST	Libercourt
ST122B	1	Bare	ST	Leforest
ST122M	1	Meadow	ST	Leforest
PE122B	6	Bare	PE	Leforest
PE122M	11	Meadow	PE	Leforest
PE122S	5	Shrub	PE	Leforest
PE122T	2	Tree	PE	Leforest
